# *“I tried to get help about my addiction but he just gave me tablets:”* male Aboriginal drug and alcohol rehabilitation clients’ experiences and preferences speaking about substance use in primary care

**DOI:** 10.1186/s12875-023-01983-0

**Published:** 2023-01-21

**Authors:** Sara Farnbach, Jamie Fernando, Joe Coyte, Matthew Simms, Maree L. Hackett

**Affiliations:** 1 National Drug and Alcohol Research Centre and The George Institute for Global Health, University of New South Wales, Sydney, NSW 2052 Australia; 2 The Glen Centre (Ngaimpe), Chittaway Point, Australia; 3 CEO, The Glen Centre (Ngaimpe), Chittaway Point, Australia; 4 South Coast Medical Service Aboriginal Corporation, Nowra, Australia; 5 The George Institute for Global Health, UNSW, The University of Central Lancashire, Preston, UK

**Keywords:** Substance-related disorders, Primary health care, Health Services Accessibility, Oceanic Ancestry Group, Australia, Aboriginal

## Abstract

**Background:**

Primary healthcare (PHC) services are crucial in supporting people who use substances. The aims of this study were to explore the experiences of Aboriginal males in NSW in treatment for substance use about speaking about their substance use with PHC staff, and their preferences for accessing PHC about their substance use.

**Methods:**

Semi-structured interviews with residential drug and alcohol rehabilitation treatment service clients. Thematic analysis was used to develop themes inductively and deductively. Two interviews were independently double coded by an Aboriginal researcher and the project was supported by an Aboriginal Advisory Group.

**Results:**

Twenty male adults who self-identified as Aboriginal participated (mean age 27 years). Half reported visiting PHC and talking about their substance use before their residential service stay. Two major themes developed: (1) speaking up about substance use or mental health problems linked with substance use, (2) ways to improve access to PHC about substance use. Although some males were offered treatment, some were not, and others had concerns about the treatments offered.

**Conclusion:**

This research highlights opportunities to improve access and to better support Aboriginal males who use substances in PHC. Focus on culturally appropriate PHC and providing staff with training around substance use and treatment options may improve access. It is important to foster culturally appropriate services, develop PHC staff knowledge around substance use, focus on therapeutic relationships and have a range of treatment options available that can be tailored to individual circumstances.

**Supplementary Information:**

The online version contains supplementary material available at 10.1186/s12875-023-01983-0.

## Background

Substance use disorder can be a chronic and relapsing condition [1, 2], often requiring ongoing or repeated treatment to achieve sustained abstinence and maintain functioning [3]. As one of the first points of contact with the healthcare system, primary healthcare (PHC) staff have opportunities to provide clinical interventions around substance use, such as behavioural counselling [4], brief intervention and withdrawal management [5]. For some people, a PHC-based brief intervention may be sufficient to reduce their substance use [6, 7]. For others who are highly dependent on substances and require more intensive treatments, PHC staff can initiate and coordinate access to treatments such as withdrawal management, psychological interventions and residential rehabilitation programs [3, 8].

Aboriginal and Torres Strait Islander (hereafter respectfully referred to as Aboriginal,) communities thrived before colonisation [9]. Social systems, roles, social order and individual and collective wellbeing were maintained via intricate kinship, spiritual and farming systems [9]. Colonisation disrupted these traditional practices [9, 10]. As a result of systemic factors and the ongoing impacts of colonisation [11, 12] and associated intergenerational trauma [13, 14], some Aboriginal and Torres Strait Islander communities today experience disproportionate harm from substance use, leading to an overrepresentation of Aboriginal and Torres Strait Islander people attending substance use treatment services [15]. Although overall there appears to be a reduction in the proportion of Aboriginal and Torres Strait Islander people who smoke and consume alcohol above the recommended amounts [15], substance use remains a national problem, accounting for approximately 6.7% of the total burden of disease and injury in the general population [16]. Nationally, it causes social and individual harm including family breakdown, violence, loss of income and imprisonment [10]. There are particular concerns about the unmet treatment needs of males, with 37% of Aboriginal and Torres Strait Islander males reporting ever using illicit substance, compared to 21% of women [15].

For PHC staff to effectively intervene for those with problematic substance use, their services must be accessible [17], and staff need to be well-equipped to facilitate discussions about substance use [18]. Limited staff time [19], multiple clinical demands [18] and limited knowledge around substance use [18], may hinder the extent to which substance use is discussed and managed in PHC. Stigma and negative attitudes held by PHC staff may also influence how likely they are to initiate discussions about substance use, or to offer screening or treatment options if concerns are raised by a patient. For instance, some staff may blame individuals, believe that a substance use disorder is a sign of weakness or a choice, or lack confidence that someone with a substance use disorder can be successfully treated [20]. Systemic barriers also exist, with many PHC services not funded specifically to provide substance use treatments.

There may be structural barriers for Aboriginal and Torres Strait Islander people when accessing PHC, including long distances to travel to services, limited culturally-acceptable options or experiences of racism or discrimination [21]. Some Aboriginal and Torres Strait Islander people may choose to access mainstream services for substance related issues [22], while others may find mainstream services unacceptable or inappropriate [23, 24] and prefer to prioritise culturally appropriate treatments [22, 25–27].

The extent to which Aboriginal and Torres Strait Islander males who use substances access PHC and discuss their substance use is unknown. This study explores the needs, experiences and preferences of Aboriginal males when accessing PHC for their substance use, before they attended residential rehabilitation (defined as community-based drug and alcohol treatment centres or residential rehabilitation services). This information will be crucial in assisting PHC staff to address substance use among Aboriginal males and for informing policies to reduce the harms from substance use. In this paper, the term Aboriginal Medical Service (AMS) is used when clients report using an AMS. Aboriginal is respectfully used to indicate the research participants self-identified as Aboriginal. If clients report having a relationship with a specific General Practitioner (GP) rather than staff at either a PHC/AMS, the term GP is used.

### Setting

The Glen Centre (Ngaimpe Aboriginal Service) is a community controlled residential drug and alcohol treatment centre on the Central Coast of New South Wales (NSW), Australia, approximately 100 kms north of Sydney. The Glen Centre provides treatment to males only (at the time of this research) and is part of the Aboriginal Drug and Alcohol Residential Rehabilitation Network (ADARRN) network of Aboriginal residential rehabilitation services in NSW. The Glen Centre’s rehabilitation treatment program runs for 12 weeks and involves group counselling, one to one counselling, self-help groups and work programs for up to 34 clients while they stay at the service. After completing the program, clients can apply for and stay on-site in a transition program which enables them to attend training and find and start employment or be discharged to a location of their choice.

## Methods

### Participants

Current male residential clients at The Glen Centre (residential rehabilitation service) aged 18 years or older and who self-reported as Aboriginal or Torres Strait Islander were invited to take part in the study.

### Aim

To understand:

1. Experiences speaking about their substance use with PHC service staff, including if care was offered as a result of these conversations; and
2. Preferences for accessing PHC for help for their substance use, before being admitted into residential rehabilitation.

### Aboriginal leadership, governance, and reflexivity

This research was conceived after one author (Aboriginal GP working in a residential rehabilitation service) noted that Aboriginal males who used substances rarely visited the PHC services where he has worked for help with their substance use. He questioned if Aboriginal males were presenting at PHC for help with concerns they had about their drug and alcohol use and whether they had preferences for how PHC access could be enhanced. This research was jointly designed and conducted by Aboriginal authors (JF and MS), Aboriginal staff at The Glen and non-Aboriginal researchers. The Glen Centre provided consent for this project. The Aboriginal authors completed all contacts with clients, contributed to data analysis and the development of the manuscript. An Aboriginal Advisory Group was convened and included staff and representatives from the residential service to oversee the research project (e.g. to guide the recruitment strategy) and also met twice during the research to review the major themes as they developed and provide feedback to the research team. To facilitate translation, a final report was provided to the Advisory Group. The active roles of the Aboriginal researchers in this research meant that they had an understanding of Aboriginal ways of knowing, being and doing and had a deep understanding of the social world in which participants were from. While there are no commercial benefits arising from this research, the intellectual property arising from this work remains with the participants and researchers (including Aboriginal researchers). The Aboriginal researchers and the Aboriginal Advisory Group shaped the research topic, approach to data collection, data collection itself and is in keeping with the philosophical origins of this project (idea generation, topic selection, data collection, analysis, interpretation, and reporting).

MS is an Aboriginal drug and alcohol counsellor who completed training in qualitative data collection and analysis and research processes (informed consent, study protocol) to develop his research capacity to complete this and future research with SF. JF is an Aboriginal GP who has completed training in qualitative data collection during his Master’s in Public Health. JF worked as a GP and in other roles at The Glen from 2015 to 2019. At the time of the research, JF worked as a GP in rural NSW. SF and MLH are non-Aboriginal researchers with experience working with Aboriginal communities and the Aboriginal co-authors on previous research projects. They have completed training in cultural competence and were provided with mentoring support from MS and JF. JC is Executive Director of The Glen Centre.

### Approach

A qualitative study using semi-structured interviews. Data were inductively and deductively analysed using thematic approach [28]. Thematic analysis [28] was chosen as it allows for a data set to be explored to identify shared meanings and experiences, therefore fits with the study goals of exploring the lived experiences of Aboriginal males about attending PHC. Data were critically analysed to focus on the strengths of participants and with recognition of the structural and systemic contexts. This approach ensured that Aboriginal people and values were prioritised in the conduct and the findings of the research.

### Sampling strategy

Semi-structured interviews were completed on site with clients at The Glen between July 2017 and December 2018. In line with a thematic approach [28], participants who were male adults and who self-identified as Aboriginal or Torres Strait Islander were purposively identified for participation in this study. During recruitment periods, a male Aboriginal drug and alcohol counsellor who had existing relationships with clients (MS or another counsellor) reviewed the current client list to identify those who appeared to meet the eligibility criteria. The counsellor then introduced the potential participant to the study and invited them to take part. If willing, eligibility was confirmed with the client and a time was arranged to complete informed consent and the research interview. Participants were reimbursed for their time with a $25 food voucher from a local supermarket.

### Data collection techniques

Interviews were conducted using an interview guide (available on request) which included questions related to the aims. The interview guide included four broad categories and 15 questions: demographic information; recent experiences seeing a GP; perceived barriers to seeing a GP; and preferences for accessing a GP after leaving The Glen Centre. The guide was pilot tested by MS and SF (using the guide SF asked MS the study questions, who provided feedback which was incorporated into the final version). The male Aboriginal drug and alcohol counsellor who contacted potential participants initially completed social yarning by following an unstructured conversation to build trust with participants [29] and then introduced them to the research topic, purpose and process. If agreeable, they then arranged a suitable time for participants to complete informed consent and ‘yarning style’ [29] interviews which followed a relaxed and interactive process broadly following the interview guide, either with one interviewer or both Aboriginal interviewers present (MS, JF). All researchers completed this project as part of their existing work commitments with managerial approval. When available, two interviewers completed interviews to facilitate participant comfort. This process also exposed both interviewers to the data as they were collected, which assisted with analysis and developing the themes. Interviews were digitally recorded and transcribed verbatim. NVivo 10 for Windows software [30] was used to manage data.

### Analysis

Following a thematic analysis approach, analysis was focused on understanding the common experiences of Aboriginal male clients. Therefore, close involvement of Aboriginal researchers was crucial to ensure cultural perspectives were central to analysis. Coding involved reading each manuscript several times [28]. The purpose of the initial readings was for the researchers to become familiar with the data and to identify the interesting and significant information in each transcript [28]. Subsequent readings were to inductively identify initial codes [28]. Transcripts were coded by SF and two transcripts were independently coded by a second coder (JF). MS, JF and SF discussed the research progress (the developing themes and their meanings and updates to the interview guide) twice during data collection and SF took notes during these conversations. The initial codes were then developed around the aims of the research. The main themes that were commonly reported were identified. The Aboriginal Advisory Group (comprising staff and representatives from the residential service) met twice to review and discuss the main themes (after stage one and at the end of the data collection). Discussions among data coders, authors and the Advisory Group were recorded using the memos function in NVivo 10 for Windows software [30]. Themes were then revised using the information recorded in the memos to ensure these discussions were reflected in the themes. Consensus around the final themes was reached among the authors and Advisory Group. This process placed Aboriginal experts’ opinions as central to analysis [10] and facilitated an in-depth understanding of the meaning of the data. After 10 interviews were completed (stage 1), the interview guide was updated based on analysis and these discussions and a 10 further interviews were completed. Data saturation was indicated with no new themes identified from the last three interviews (interviews 18–20).

### Ethics

This research was approved by the Aboriginal Health and Medical Research Council (1260/17) and the Board at The Glen. The manuscript was provided to AHMRC for approval. This research was conceived, designed and conducted in line with the principles set out in the *Ethical conduct in research with Aboriginal and Torres Strait Islander Peoples and communities: Guidelines for researchers and stakeholders *developed by the National Health and Medical Research Council [31]. This research is reported following the Consolidated criteria for reporting qualitative research checklist [32] and the Consolidated criteria for strengthening reporting of health research involving Indigenous peoples [33].

## Results

Interviews were conducted with 20 males (Table 1), typically taking 12-45 min, resulting in approximately 245 min of recorded interview data. All males who were invited, participated in the research. All participants self-reported as Aboriginal. No Torres Strait Islander males took part. The average age was 27 years. Most males were from rural, remote or regional NSW. The two major themes included: 1) Speaking up about substance use or mental health problems linked with substance use, and 2) Ways to improve access to PHC for substance use. Sections where results are based on a subgroup of 10 participants are identified in the results (10 participants reported having recently seen a GP). All remaining sections include results for all 20 participants.

**Table 1:** Characteristics of the Aboriginal residential drug and alcohol clients who completed interviews

Participant characteristics	*N* = 20
***Ethnicity***	
Aboriginal	20
Torres Strait Islander/other	0
***Age (mean, years)*** 27 years	
19 to 39 years	
***Usual residence***^***a***^	
Rural, remote or regional	15
Sydney/Newcastle metropolitan	5
***Full or part time paid work (in the last 12 months)***	
Yes	12
No	8
***Contacted a primary healthcare staff about substance use/mental health, prior to The Glen***	
Yes	10
No	10
***Main drug of choice***	
Ice/methamphetamine	11
Cannabis	2
Alcohol	2
Other	3
Not reported	2

^a^All participants resided in New South Wales, Australia

### Speaking up about substance use or mental health problems linked with substance use

Ten participants reported that before their current stay at The Glen they spoke with a staff member at a PHC service about concerns they had about their substance use or mental health (this section includes data from these 10 participants). Approximately half of these clients who spoke to a GP about their substance use reported that they had an established relationship with the GP, which generated trust. Some of these participants perceived that their substance use was linked with mental health problems, such as anxiety, sleep problems, hearing voices, anger management or depression, and spoke of these concepts together:

So I went down there [to the PHC service] and said to them “… I need to see them about my drug use and a bit of depression and that…”

25 years, #3

These clients reported speaking to GPs, Aboriginal Health Workers or receptionists when they were booking an appointment about their substance use or mental health problems.

Clients initiated these conversations when they perceived that their substance use was causing them serious problems such as experiencing paranoia, extreme anger or they were at crisis point:

Well last time I went there … I told him [GP] I was hearing things and I was that paranoid I got four people following me around.

28 years, #1

One participant reported telling reception staff at an AMS that he wanted to see somebody about his substance use and mental health, and he was told he would have to wait for the next appointment in two hours, which he was unable to do in his current state. None described being screened for substance use by PHC staff, although the occurrence of screening was not explicitly explored during interviews.

#### Conversations resulted in receiving or not receiving treatment

Some clients reported that these conversations resulted in them receiving the treatment which they sought (e.g. the clients had a Mental Health Care Plan established or were referred to a substance use treatment service) and that this was a positive outcome.

Conversely, there were many other clients who despite wanting to be referred for treatment, were not referred. One participant, after explicitly asking for help because he reported he was unable to stop using drugs, was told by the GP that he didn’t need treatment:

I said, mate, “I don’t like this life, I don’t like who I am”, I’m crying aloud, and he goes, “morally you’re halfway there. I don’t think you need rehab … you’ll be right, just tough it out”.

34 years, #15

When prescribed medication (e.g. to reduce anxiety or assist with sleep), many clients were reluctant to take it because they didn’t feel it was the type of treatment they needed:

Yeah, I already spoke to him [GP] about that sort of stuff [substance use]. I tried to get help off him … but he just gave me tablets.

20 years, #8

Others reported previously experiencing problematic side effects from medication or that they thought that a more detailed assessment was needed to fully understand their problem and a prescription alone did not address their situation:

The first AMS I went to they were just asking about my sleep. And they tried to get me on tablets, which I told them I didn’t want to [sic]. I went there for depression as well. And I didn’t want to swap a drug for a drug.

25 years, #3

#### Concerns about talking to a GP about substance use

Some clients reported concerns talking to a GP about their substance use because they had concerns that they would receive bad news about their health, that they would be judged, due to stigma associated with substance use or that their children might be taken away as a result of these discussions:

The first time I opened up to a couple of doctors ‘cause I was trying to get help, I was just so lost. And he’s going, “no you’re right”. But in saying that too, I didn’t explain it ‘cause I was scared to tell him I had a drug problem.

34 years, #15

These clients were not referred for treatment. In contrast, one participant reported that because their GP knew them and their family, he chose not to speak about his substance use because he was concerned that his family may find out. It was common for participants to report that substance use caused their lives to be chaotic or unmanageable, and consequently they stopped visiting PHC:

But the last couple of years I haven’t been seeing him [the GP]. I’ve just been on drugs and that, just didn’t care about my life. [I] didn’t care about my health.

28 years, #1

### Ways to improve access to PHC about substance use (analysis includes data from all participants)

For many clients, fostering an environment that was supportive of their needs, by visiting their regular GP, Aboriginal staff member or having a support person (family member or friend) to attend appointments with them, may increase their use of PHC. The participants also discussed improved communication between the PHC service and themselves, compensation for their time (financial or gift) and linking PHC with cultural activities as potential activities that could assist with access to PHC.

#### Access to Aboriginal staff and the same staff member at PHC services

Some clients indicated that having access to a staff member who was also Aboriginal would be useful because they could assist with communication between themselves and PHC staff and help clients to understand health information. In addition, three reported that an Aboriginal staff member would understand their situation without judgement, which was important because some had felt judged by non-Aboriginal staff at mainstream health services:

Cause they’re [non-Aboriginal staff] just not on the same level. They sit there and they like they just don’t understand, they don’t realise the causes of things, the reasons why you do things. They just look at what you are now and who you are now and the choices you make now, let alone [understanding] what led up to all of that.

25 years, #11

Many reported that they would prefer to visit the same GP who they trusted on an ongoing basis because some parts of their pasts were difficult to discuss, and they didn’t want to repeat their story to a new GP at each appointment:

I've had mental health issues, drug issues, alcohol issues, and some of these things are hard to talk about. And sometimes you feel like you've just got to pour your heart and soul out to a stranger. That's not the easiest thing to do.

28 years, #9

When comparing AMSs with mainstream services, some clients reported that AMSs had a more people-friendly and culturally appropriate approach, meaning staff had more time to spend with clients to understand their needs than at mainstream services and that this was of benefit to them. One participant reported that AMS staff were less judgemental of his circumstances than mainstream PHC staff. Conversely, some clients identified some benefits of mainstream PHC services including that they often have more appointments available, which was important when they needed one urgently.

#### Building on existing cultural and community strengths via ‘support groups’, men’s groups, families and communities

Some clients reported that they knew that some AMSs were linked with support groups or men’s groups that had a focus on culture, such as cultural dancing or painting. For these clients, cultural programs provided opportunities for them to link their treatment with their cultural connections, and this was an important part of their recovery. Although many clients were aware that these groups existed, none reported being part of a group before they came to The Glen, and were unaware how to join such a group. About half of the clients were interested in joining a group after they leave The Glen:

I hope I can find some of my mob that’s doing an activity or something. I’ve always wanted to go through the passage of rites, like from child to man.

22 years, #13.

Most clients were able to identify at least one family member or friend who was their support person. This support from within their community was frequently reported by clients to be important because it prompted them into positive action around their health and may help them to attend PHC appointments.

#### Enhancing experiences when accessing PHC services

Clearer communication between the PHC service and themselves was important to encourage clients to make initial visits and on an ongoing basis. Some clients suggested that PHC sent appointment reminders via text-message as this may be a helpful prompt for them to attend appointments. Others reported that having an initial appointment with a new GP in their homes (as a home visit) would help them to build trust with the GP and increase the likelihood that they would  attend subsequent appointments.

A few clients reported that some AMSs provide clients with a jersey or a voucher after completing health checks to compensate for their time. For these clients, this compensation motivated them to attend appointments or alleviated financial stress as it helped them to prioritise their health over other life issues:

We might have a doctor’s appointment but have no money for that day and we put other priorities instead of going to the health [service] like trying to get loans, trying to survive. They [community members] want to go there ‘cause they get the voucher … they don’t have to worry about the rest of their day, it makes it open for them to go to the doctor’s appointment to get health checks.

25 years, #11

## Discussion

To our knowledge, this is the first research exploring the experiences of Aboriginal males speaking about their substance use with staff at PHC services. Although it is encouraging that half the males who took part had spoke up and sought help from PHC at some stage before entering residential rehabilitation, suggesting that they were motivated to reduce or stop their substance use, it is concerning that many of these males did not receive treatment or had concerns about the type of treatment they were offered. Many males made suggestions about ways that access to PHC for substance use could be improved.

Although some participants were offered treatment after conversations about their substance use with PHC staff, many were not, and some were told they did not need treatment, despite the participants expecting or asking for it. Staff-related factors such as heavy workloads [18], limited time [19] and skills [18], or stigma, values and negative beliefs held by staff around the likely success of treatment for people who use substances may contribute to the extent to which males report their substance use [34] and are offered treatment [20]. By providing ongoing education opportunities to PHC staff around the substance use and management options, PHC access may be improved by increasing staff knowledge, skills and awareness and reducing stigma and negative beliefs [34]. Although there is limited research around effective PHC-based strategies to reduce substance related harms among Aboriginal populations [5, 35], there is growing international evidence around the role of culturally acceptable PHC based substance use programs. Brief interventions [36], screening [37] and contingency management [38] in Australia, and motivational interviewing incorporating culturally relevant treatments (e.g. talking circles and sweat lodge ceremonies [39], drawing on spiritually and family/clan relationships as motivation and delivered by counsellors fluent in the native language) [40], in America, all have promise. Given that some Aboriginal people prefer to prioritise culturally appropriate treatments [22, 25–27] and the value placed by participants in this study on community and cultural connections, this focus on culturally relevant programs is warranted and continued efforts in this area may enhance PHC services’ role in identifying and supporting Aboriginal males who use substances.

As has been found previously with Aboriginal women [41], some males did not disclose the extent of their substance use to GPs for a variety of reasons, including stigma, concerns about receiving bad news, and understandably because they preferred to see the same GP who they trusted on an ongoing basis. Trust between themselves and the GP was also identified as important to participants. These findings further highlight the importance of providing PHC staff with training and information around substance use and available treatment options. PHC needs to be a safe place where people develop trust with PHC staff so they feel comfortable to speak openly about their concerns, without judgement or welfare repercussions, and can expect to receive or be referred for culturally appropriate treatment when it is requested.

Cultural support and culturally-appropriate approaches are important elements of residential drug and alcohol treatment services for Aboriginal clients [25–27], and of AMSs, many of which have a focus on culturally informed service delivery [42]. Participants in this study described culturally-appropriate PHC services as those with staff who have time to understand their needs, with Aboriginal staff and where they did not feel judged. Problematic substance use arises from a range of complex social and historical determinants [43], including trauma [10], and these findings indicate that clinicians who understand these causes and with whom males do not feel judged by is important when discussing and treating substance use. Recognising the importance of culturally-appropriate services, the peak organisation for Aboriginal residential rehabilitation services in NSW (ADARRN) has developed a model of care which incorporates evidence-based practice with embedded cultural values [27]. The model of care includes six core components and activities to operationalise each core component that can be standardised in any Aboriginal residential rehabilitation service [27]. One of the components is cultural healing, meaning that each service can tailor cultural activities according to their context and circumstances [27]. The findings of this research indicate that this focus on culturally-appropriate service delivery is important to clients, and access to mainstream PHC services by Aboriginal males may be improved with increased focus culturally-appropriate service delivery.

Although access to Aboriginal staff at PHC services was identified as important (because they may assist with communication and were less likely to be judgemental), staffing continuity between appointments was also important because clients did not want to repeat their story at each appointment. This is understandable, given the sensitive nature of substance use and its links with trauma [10]. Together with the diverse opinions expressed by the participants about their preference to visit a mainstream GP or AMS, these findings highlight that as well as focusing on culturally-informed services, a range of options and services should be available, treatment should be individually tailored and culturally safe and relevant activities and groups post residential rehabilitation should be encouraged.

A strength of this research is the active role of the Aboriginal researchers in conception, design, analysis and reporting of this project. Another strength is the existing relationship between participants and the interviewers which may have encouraged open and honest conversations. Although these relationships may have caused clients to report more positively their attendance at PHC, we believe this approach facilitated the research by encouraging participation as all eligible males who were asked, took part. Use of the semi-structured interview guide meant that detailed information about the experiences of participants was gained. The interview guide was updated based on the responses of early participants and the framework was revised as interviews were coded. This research may be limited by recall bias as it relied on participants recalling difficult events from a long time ago for some. This is a small research study including 20 males from one Aboriginal residential rehabilitation service. All were from NSW and all were in treatment. There may be differences between the participants and those who were unable or unwilling to take part, including clients who had not yet accessed treatment, who had dropped out of the program early or were not asked, which may have led to selection bias. As such these findings may not be generalisable to other contexts or other residential rehabilitation services. Further work with males not in treatment would provide insights into the experiences of those not yet in treatment or those with more moderate use.

This research highlights the importance of fostering a PHC environment where discussions about substance use are likely to occur. To support PHC services to provide such care, structural issues should be addressed such as making funding available to deliver care around substance use. Staff should be offered training to build knowledge around effective strategies to support people who use substances, and working with Aboriginal people. Further research is needed into the impact of models such as the standardised model of care in Aboriginal residential rehabilitation services [27], in order to identify the extent to which these can improve outcomes for Aboriginal clients and if they can be extended to other settings, such as PHC. This research provides an example of a co-designed research project, which was initiated by Aboriginal staff and completed in partnership with non-Aboriginal researchers.

## Conclusions

The challenges the Aboriginal males faced when speaking about their substance use with the hope of accessing treatments is concerning and highlights the need for developing PHC staff knowledge around substance use treatments, delivery of culturally-appropriate care, focusing on developing ongoing therapeutic relationships and having a range of treatment options available that can be tailored to individual circumstances. This research is encouraging as it shows that many Aboriginal males in treatment for substance use had sought help from PHC, demonstrating a keen interest in improving their health.

## Supplementary Information

**Additional file 1.**

## Data Availability

The datasets generated and/or analysed during the current study are not publicly available due to the sensitive nature of the datasets and personal information provided by participants, but are available from the corresponding author on reasonable request.
